# A unique microRNA profile in end-stage heart failure indicates alterations in specific cardiovascular signaling networks

**DOI:** 10.1371/journal.pone.0170456

**Published:** 2017-03-22

**Authors:** Sathyamangla V. Naga Prasad, Manveen K. Gupta, Zhong-Hui Duan, Venkata Suresh K. Surampudi, Chang-Gong Liu, Ashwin Kotwal, Christine S. Moravec, Randall C. Starling, Dianne M. Perez, Subha Sen, Qingyu Wu, Edward F. Plow, Sadashiva Karnik

**Affiliations:** 1 Department of Molecular Cardiology, Lerner Research Institute, Cleveland Clinic, Cleveland, Ohio, United States of America; 2 Department of Computer Sciences, University of Akron, Akron, Ohio, United States of America; 3 Department of Molecular Virology, Immunology and Medical Genetics and Comprehensive Cancer Center, Ohio State University, Columbus, Ohio, United States of America; 4 Department of Cardiovascular Medicine, Heart and Vascular Research Institute, Cleveland Clinic, Cleveland, Ohio, United States of America; Texas A&M University Health Sciences Center, UNITED STATES

## Abstract

It is well established that the gene expression patterns are substantially altered in cardiac hypertrophy and heart failure, however, less is known about the reasons behind such global differences. MicroRNAs (miRNAs) are short non-coding RNAs that can target multiple molecules to regulate wide array of proteins in diverse pathways. The goal of the study was to profile alterations in miRNA expression using end-stage human heart failure samples with an aim to build signaling network pathways using predicted targets for the altered miRNA and to determine nodal molecules regulating individual networks. Profiling of miRNAs using custom designed microarray and validation with an independent set of samples identified eight miRNAs that are altered in human heart failure including one novel miRNA yet to be implicated in cardiac pathology. To gain an unbiased perspective on global regulation by top eight altered miRNAs, functional relationship of predicted targets for these eight miRNAs were examined by network analysis. Ingenuity Pathways Analysis network algorithm was used to build global signaling networks based on the targets of altered miRNAs which allowed us to identify participating networks and nodal molecules that could contribute to cardiac pathophysiology. Majority of the nodal molecules identified in our analysis are targets of altered miRNAs and known regulators of cardiovascular signaling. Cardio-genomics heart failure gene expression public data base was used to analyze trends in expression pattern for target nodal molecules and indeed changes in expression of nodal molecules inversely correlated to miRNA alterations. We have used NF kappa B network as an example to show that targeting other molecules in the network could alter the nodal NF kappa B despite not being a miRNA target suggesting an integrated network response. Thus, using network analysis we show that altering key functional target proteins may regulate expression of the myriad signaling pathways underlying the cardiac pathology.

## Introduction

Heart failure is a major cause of morbidity in the elderly across the globe. End-stage heart failure is characterized by significantly perturbed neuro-hormonal and mechanical (hemodynamic) stimuli to the heart. The altered pathological signaling leads to remodeling of the heart with adaptive to maladaptive hypertrophy transitioning into dilated cardiomyopathy (DCM). DCM is the most common and well documented outcome of various deleterious stimuli the heart perceives [1] leading to heart failure. The myocytes undergo remodeling through activation of intracellular signaling pathways involving a cross-talk between deleterious and compensatory signaling pathways. Despite significant advances in identifying genes and signaling pathways, the overall complexity of the hypertrophic remodeling suggests the involvement of global regulatory mechanisms modulating signaling networks. Increasing body of evidence suggests that small non-coding RNAs such as microRNAs (miRNAs) could play a key role in modulating global signaling networks as they are involved in diverse processes including cell proliferation, cell death, metabolism and neuronal patterning [2].

miRNAs are small non-coding RNA molecules approximately 22 nucleotides in length which act as post-transcriptional regulators of gene expression [2–4]. Individual miRNAs are known to regulate the expression of multiple genes and have been extensively investigated in cardio-vascular disease using numerous cellular and animal models that have been reviewed extensively [5–7]. However, validation of specific miRNA target genes and identification of the affected signaling pathways have not yet been extensively studied and/or discussed as many of these studies have assessed effects of miRNA on single target. In this regard multiple studies have shown that miRNAs play an important role in cardiac development [6, 7], progression towards heart failure including cardiac remodeling [8, 9] and DCM [10, 11]. The small targeting sequence of miRNA theoretically allows for binding of miRNAs to many gene products ultimately regulating the expression of target genes in diverse signaling pathways. Therefore, miRNAs could potentially play a significant role in global regulation of signaling networks during remodeling and transition to heart failure. Given the knowledge that miRNAs are altered in heart failure and by regulation their target proteins, it would be critical to assess their roles in modulating global signaling networks in addition to their role in regulating expression of specific gene products. More importantly, bioinformatics data suggest that each single miRNA potentially regulates very large sets of genes (as hundreds of putative gene targets are known) [12, 13]. This highlights the potential influence of miRNAs on almost every metabolic and regulatory pathway considering that miRNAs potentially regulate more than 60% of mammalian mRNAs [14, 15]. Our current study was designed to elucidate the global regulation of the signaling networks by the unique miRNA pattern in end-stage human heart failure. Therefore, the goals of our study were: a) to measure the changes in expression of 288 human miRNAs using a custom micro-array hybridization platform, b) use an independent set of human heart failure samples to validate this data set, c) through an *in silico* strategy of bioinformatics delineate canonical/functional pathways regulated by genes that are potential targets of miRNAs in heart failure in order to provide a global picture of the molecular network and nodal molecules regulated by these miRNA, d) analyze the expression pattern of target nodal molecules using the publicly available gene expression databases (cardio-genomics data base) from end-stage human heart failure, immunoblotting for critical nodal molecules from the same set of patient samples to demonstrate inverse correlation between protein expression and miRNAs level and f) use surgical mouse model of heart failure to assess key nodal molecules in the signaling pathway. Use of systems biology approach provides for a better understanding of the underlying regulation of multiple genes and pathways involved in this complex pathology of heart failure. Comprehensive studies on signaling networks could provide an idea of global pathways that could be altered in response to novel therapeutics strategies.

## Materials and methods

### Patient samples

Tissue from the left ventricular free wall was obtained from explanted hearts of transplant recipients at the Cleveland Clinic with a diagnosis of DCM. The non-failing control hearts were obtained from unmatched donors whose hearts were not suitable to transplantation despite normal ventricular structure and function as measured by echocardiography. The hearts were arrested and transported in ice-cold, oxygenated cardioplegic solution [16]. Once in the lab, the tissue was flash frozen in liquid N_2_ and stored at -80°C. All protocols for tissue procurement and procedures carried out on the tissues was in performed in compliance with institutional guidelines for human research and approved by the Cleveland Clinic Institutional Review Board.

### Ethics statement

End-stage human heart failure hearts were obtained from patients receiving heart transplants at the Cleveland Clinic. All the hearts from both failing as well as non-failing were obtained following a signed consent for the use of the heart tissue in molecular and biochemical studies. These heart samples were obtained in accordance with and in compliance with the approved Cleveland Clinic Institutional Review Board protocol of Cleveland Clinic. The Cleveland Clinic Institutional Review Board with Federalwide Assurance (FWA 00005367) which is accredited by the National Association for the Accreditation of Human Research Protection Programs. INC. The protocol # IRB 2378 titled “Functional, Biochemical and Molecular Correlates of End-stage Human Heart Failure” and is active till 1/21/2017.

### RNA isolation

RNA was isolated as previously described [16]. Briefly, 100 mg of left ventricular tissue was homogenized using TRIZOL (INVITROGEN) reagent and the homogenized samples were incubated at room temperature for 5 min. Chloroform was added to the samples, vigorously mixed and incubated at room temperature for 5 min. Following incubation, the samples were centrifuged at 12,000 X *g* for 15 min at 4°C. RNA was precipitated from the aqueous phase by addition of isopropanol to a fresh tube containing the supernatant aqueous phase. The integrity of the RNA was tested by spectroscopic analysis and by running samples on a denaturing formaldehyde gel.

### Target preparation and array hybridization

Target preparation and array hybridization were carried out as previously described [17]. Briefly, five μg of total RNA was added to biotinylated oligonucleotide primer. Following incubation first strand was synthesized using Superscript II RNAseH^-^ reverse transcriptase. After the synthesis of the first strand, the reaction was incubated at 65°C to denature the RNA/DNA hybrids and degrade RNA templates. The labeled targets were then used for chip hybridization. Hybridization was carried out on the miRNA microarray (Ohio State Comprehensive Cancer Centre, version 3.0) containing 627 probes for mature miRNA corresponding to 288 different human miRNAs spotted in quadruplicates. Often, more than one probe set is present for a given mature miRNA and there are quadruplicate probes corresponding to most precursor miRNAs. The detection of biotin-containing transcripts were carried out by streptavidin-Alexa Fluor 647 conjugate and scanned images (Axon 4000B) were quantified using the GenePix 6.0 software (Axon Instruments).

### miRNA microarray data and miRNA target prediction

Microarray images generated by the array hybridization were analysed by GenePix Pro. Average values of the replicate spots for each miRNA were background subtracted, normalized and subjected to further analysis. Global median normalization and Lowess normalization of the heart microarray data was carried out using the BRB ArrayTools [17]. Following the identification of differentially expressing miRNAs, the predicted targets for these differentially expressed miRNAs were identified using TargetScan 3.1 and PictTar databases [18–20]. Use of the recently released TagetScan 4.2 did not significantly alter the nodal molecules identified in this study. The requirement for analysis using the different databases was based on the need to encompass all the potential targets as they are built slightly different algorithms. We have used results of predicated targets from TargetScan database to carry out pathways and network analysis.

### Network analysis

A data set containing genes and the corresponding expression values was uploaded into the Ingenuity Pathways Analysis Network^™^ application. The dataset molecules of our interest (predicted targets of altered miRNA) which interact with other molecules in the Ingenuity’s knowledge base are identified as network eligible molecules. Network eligible molecules serve as “seeds” for generating networks. Network eligible molecules are combined into networks that maximize their connectivity in the Ingenuity’s knowledge base. A defined network is limited to a maximum of 35 molecules and the additional molecules from the Ingenuity’s knowledge base are used to connect networks resulting in large merged networks. Networks of these focus genes based on their significance of cut off were then algorithmically generated based on their connectivity. A network pathway is a graphical representation of the molecular relationships between genes/gene products. Genes or gene products are represented as nodes, and the biological relationship between two nodes is represented as an edge (line). All edges are supported by at least one reference from literature, textbook or from canonical information stored in the Ingenuity Pathways Knowledge base. Nodes are displayed using various shapes that represent the functional class of the gene product. Edges are displayed with various labels that describe the nature of the relationship between the nodes (i.e., P for phosphorylation, T for transcription etc.).

### Canonical pathway and functional analysis

Canonical pathway analysis was carried out using the Ingenuity Pathways Analysis (IPA) library of canonical pathways by uploading the data set of predicted targets for the significantly altered miRNAs in DCM to the IPA server. These target genes (focus genes) were analysed for over-representative canonical pathways in control and diseased human samples. Functional analysis of a network identified the biological functions that were most significant to the genes in the network. The network genes associated biological function/disease state in the Ingenuity knowledge [12] base was considered for analysis.

### Cardiac microarray expression database

To test for expression trends in the target gene sets, microarray data from cardio-genomics database for normal and idiopathic heart failure were used (http://cardiogenomics.med.harverad.edu/project-detail?project_id=229). The raw data were reprocessed using the GCRMA algorithm which is a three step function implemented in the GCRMA package (version 2.8.1) of the Bio-conductor open source library (version 2.5.0) (http://www.bioconductor.org/). Steps include correction of perfect match probe set expression signals for optical noise and non-specific binding using probe sequence information, followed by quantile normalization to smooth individual probes intensities. Finally, expression values were summarized by the robust multi-chip model fit using median polish. The summarized probe set expression values were subsequently fit to a linear correlation analysis model.

### Quantitation of miRNA

Total RNA was isolated by Trizol reagent (Invitrogen) and RNA concentration was determined by spectrophotometer. 2 μg of RNA was used for reverse transcription with reverse transcription kit (Applied Biosystems) using specific primer sets as supplied with the Taqman assay kits for individual miRNAs. Quantitative real time PCR was performed using cDNA with Taqman real time mix in a BioRad iCycler machine. The fold change in the miRNA levels was determined using RNU6B as an internal control. ΔΔCt method [21] was used to compare the DCM samples against the normal controls. The 2^-ΔΔ*CT*^ method was used as relative quantification strategy for quantitative real-time polymerase chain reaction (qPCR) data analysis.

### Western immunoblotting on patient samples, mouse hearts, HEK 293 and HL-1 cells

End-stage human heart failure samples (100 mg) and mouse hearts (sham and TAC) were homogenized using Polytron homogenizer in 1.5 ml of lysis buffer and HEK 293 or HL-1 cells were lysed in 100 μl lysis buffer (1% NP-40, 10% glycerol, 137 mM NaCl, 20 mM Tris-Cl (pH 7.4), 1 mM PMSF, 20 mM NaF, 1 mM Na-pyrophosphate, 1mM Na-orthovanadate and 2 μg/ml each of aprotinin and leupeptin) and centrifuged at 38,000 X *g* for 25 min. The supernatant myocardial lysates (180 μg) were resolved on SDS-PAGE gel and immunoblotted with various primary antibodies, anti-RB1 (1:250, BD Biosciences), anti-ERBB2 (1:500, Santa Cruz), anti-STAT3 (1:1000, Santa Cruz), total anti-NFκB (1:500, Santa Cruz), anti-HDAC4 (1:300, Genescript), anti-COL1 (1:500, ROCKLAND), anti-MMP2 (1:250, BIOMOL INTL), anti-TIMP2 (1:250, Ab cam), anti-EZH2 (1:1000, UPSTATE), anti-E2F3 (1:500, Ab cam), anti-SLC7A1 (1: 500, Novus Biologicals), anti-HDGF (1: 1000, Santa Cruz), anti-BCL2 (1:500, Santa Cruz) and anti-β-actin (1:200, Santa Cruz). Appropriate secondary antibody (1:2000) was used and detection carried out using enhanced chemiluminescence [12] (Amersham).

### Cell culture, transfection and northern blot analysis

Cell culture and transfection studies were carried out as previously described [22]. Briefly, HEK 293 cells were maintained in minimal essential medium (MEM) supplemented with 10% fetal bovine serum and penicillin-streptomycin at 37°C. Cells will be seeded at a density of ~1–3 x 10^5^ cells/ 35 mm dish and transfected at 60–70% confluence using FUGENE6 (ROCHE). Mature human miRNA-7 (5’- UGGAAGACUAGUGAUUUUGUUGU-3’) was cloned into pRNAT.CMV3.1/Neo (Genscript Corporation) between the Bam H1 and Afl II sites which also carries cGFP (coral GFP) for convenient tracking of transfection efficiency. Human miRNA-7 expressing pRNAT.CMV3.1 was transfected into HEK 293 cells. For ERBB2 expression analysis, HEK 293 cells were transfected with scrambled control (100 ng), miRNA 7 (100 ng) or antagomir 7 (100 ng). HL-1 cells were maintained in Claycomb Media supplemented with serum and were seeded ~1.5 x 10^6^ cells/35 mm dish. The cells were transfected at 80% confluence using Lipofectamine (Invitrogen) with allstar scrambled control (300 ng) or miRNA 214 (150 ng) along with miRNA 378 (150 ng) or miRNA 214 (150 ng) along with antagomir for miRNA 378 (150 ng). 48 hours post transfection, cells were harvested and lysed in lysis buffer (as described above) the lysates were resolved on SDS-PAGE gel for western immunoblotting. Northern blot analysis was carried out as previously described (2). Briefly, 48 hours post-transfection cells were lysed in TRIZOL reagent as per manufacturer’s instructions. 10 μg of RNA was size-fractionated by denaturing formaldehyde gel electrophoresis, transferred to nylon membrane by capillary action and cross-linked using UV cross-linker. The membrane was stained with 0.5% methylene blue to visualize the transferred RNA onto the nylon membrane and to check for equal loading across lanes. The membranes were destained with diethylpyrocarobonate (DEPC)-treated water. After de-staining the membrane was hybridized with [^32^P] labeled mature miRNA-7 cDNA. Following hybridization, the filter were washed under stringent conditions and transcripts visualized by autoradiography.

### In vivo pressure overload hypertrophy; Transverse Aortic Constriction (TAC)

C57BL/6 mice 8–12 weeks of age underwent microsurgical procedure of transverse aortic constriction (TAC) to induce pressure overload hypertrophy and cardiac remodeling in mice as described previously [23]. Briefly, following anesthesia with ketamine and xyalzine, the mouse was connected to a rodent ventilator. The chest cavity was entered in the second intercostal space and the transverse aorta between the right (proximal) and left (distal) carotid arteries was isolated. Transverse aortic constriction (TAC) was performed by tying a 7–0 nylon suture ligature against a 27-guage needle. The needle was promptly removed to induce pressure overload mediated cardiac remodeling. After aortic constriction, the chest was closed, pneumothoras was evacuated and the mouse was extubated, and allowed to recover from anesthesia. Sham operated animals underwent the same except for the aortic constriction.

### Ethics statement

Mice following surgery were monitored for pain post-TAC and normally administered bupivacaine to alleviate pain. After 0, 1, 2, 3, 4, 7 and 12 days of aortic constriction, mice were exsanguinated under anesthesia using ketamine/xylazine and hearts were rapidly excised, individual chambers were separated, weighed, and frozen in liquid N_2_ for miRNA analysis or for western immunoblotting. Animals were handled according to the approved protocol and animal welfare regulations of the institutional review board at Cleveland Clinic. The protocol was approved by AALAC accredited animal welfare IACUC board of Cleveland Clinic and the approved protocol # ARC 2014–1317 that is active till October 2017.

### Statistical methods

#### Computational analysis of miRNA microarray data

Microarray images were analyzed by GenePix Pro wherein average values of the replicate spots for each miRNA were background subtracted and normalized. Global median normalization and Lowess normalization of microarray data was carried out using the BRB ArrayTools [17]. The probes with over 70% missing data were excluded from further analysis. Differentially expressed miRNAs between control and dilated cardiomyopathic samples were identified by using “*t test*” procedure within significance analysis of microarrays (SAM). Furthermore, Lowess normalization was carried out to analyse differentially expressed miRNAs in control versus diseased state. Differentially expressed miRNAs were then used for identifying predicted targets.

#### Network analysis

Ingenuity’s knowledge base was used to connect networks resulting in large merged networks. Predicted targets for the altered miRNAs were mapped to the corresponding gene object in the Ingenuity knowledge base. A specific value for cut-off of 1.5 was set to identify genes whose expression was significantly differentially regulated. These genes, called focus genes, were overlaid into a global molecular network developed from the information contained in the Ingenuity Pathways Knowledge Base.

#### Canonical pathway and functional analysis

Significance of association between these genes and the canonical pathway was measured in two ways: a) A ratio of the number of genes from the data set that map to the pathway divided by total number of genes that map to the canonical pathway is displayed and b) Fischer’s exact test was used to calculate a “p” value determining the probability that the association between the genes and the canonical pathway is explained by chance alone. The network score is based on the hyper-geometric distribution and is calculated with the right-tailed Fisher’s Exact Test and is represented as a negative log of this p-value. For example, a network of 35 molecules has a Fisher Exact Test p-value of 1 x 10^−6^, the network’s score = -log (p-value) = 6 [24].

#### miRNA quantitation/Real time PCR analysis/Western immunoblotting

In our studies with human samples as well as mouse hearts, we have used RNU6B as an internal control. We have used the ΔΔCt method [21] used to compare the DCM samples against the normal controls. Importantly the 2^-ΔΔ*CT*^ method can be effectively used for relative quantitative assessment of the real-time polymerase chain reaction (qPCR) data wherein, we have used threshold cycles (*CTs*) generated by the qPCR system for calculation. For the miRNA analysis of the TAC mice, the 2^-ΔΔ*CT*^ was used to compare with the Sham mice as control. Following this calculation and normalization the fold changes for each time point for all the mice were analyzed with control (Sham) using an unpaired *t test* to assess for significance wherein, *p<0.05 over control was significant. Furthermore, ΔΔCt method was used for the human non-failing and failing DCM samples wherein, *p<0.05 was considered significant. Densitometry was performed on the NFκB and loading controls (actin for human studies and GAPDH for mice studies) following western immunoblotting. Fold over respective controls are presented for various samples and the data is presented as mean ± SEM. Unpaired *t test* was performed to compare between non-failing and failing (DCM), Sham and TAC or vector control to miRNA transfections wherein, *p<0.05 was considered significant.

## Results

### DCM patient population characteristics and associated miRNA signature

To determine signaling networks/canonical pathways that are altered in response to changes in miRNA expression pattern in human heart failure, we used mRNA from 70 patient samples including 20 non-failing and 50 end-stage heart failure patient samples diagnosed with DCM. All heart failure patients were in New York Heart Association (NYHA) class IV category with left ventricular ejection fractions < 15% while, non-failing patients had ejection fraction > 61% (Table 1). The average age of the patients was 52.5 ± 3 yrs wherein the mean for DCM patients was 51 ± 2 yrs and for non-failing was 54 ± 1 yrs. The non-failing patients have relatively normal ventricular function as measured by echocardiographic function. The studies were gender and race independent (Table 1). The miRNA profiling was carried out as a two-step strategy wherein the first screen was performed using a validated [17] custom miRNA microarray platform containing 5760 miRNA probes (encompassing 288 human miRNAs) by classical hybridization methodology followed by validation of the altered miRNAs using RT-PCR on an independent set off patient samples (S1 Fig). Identification of altered miRNAs in end-stage human heart failure will allow us to build signaling networks that would be affected based on the target molecules.

**Table 1:** Patient demographics.

**Failing Hearts (n = 20)**				
**Age**	**Sex/Drug Therapy**	**Race**	**EF**	**Cause of Death**
51±2	11F, 9M Acute -NE, DA, Other Chronic–“HTN meds”	19 W, 1 B	61 ± 2	14 CVA, 2 MVA,1 GSW, 1 Trauma, 1 Anoxia
**Failing Hearts (n = 50)**				
**Age**	**Sex/Drug Therapy**	**Race**	**EF**	**Diagnosis**
54±1	17 F, 33M DIG, DOB, AMIO ACEI, BB	42 W, 8 B	15±1	50 DCM

Abbreviations: Non-Failing Hearts: F = female, M = male; W = white, B = black; EF = left ventricular ejection fraction measured prior to explant; CVA = cerebrovascular accident, MVA = motor vehicle accident, GSW = gunshot wound; Drug Therapy Acute = treatment in the emergency room or intensive care unit prior to brain death: NE = norepinephrine (n = 9), DA = dopamine (n = 12), OTHER = epinephrine, pitressin, phenylephrine, labetolol, lisinopril (n = 1 or 2); Drug Therapy Chronic = drugs taken by patients prior to admission, as reported by family members (n = 6).

Failing Hearts: F = female, M = male; W = white, B = black; EF = left ventricular ejection fraction measured prior to explant; DCM = dilated cardiomyopathy (pre-transplant diagnosis); Drug Therapy lists those drugs taken by over 25% of patients in the group, DIG = digoxin, DOB = dobutamine, AMIO = amiodarone, ACEI = angiotensin converting enzyme inhibitor (usually lisinopril), BB = beta adrenergic blocker (metoprolol or carvedilol).

**Fig 1:**
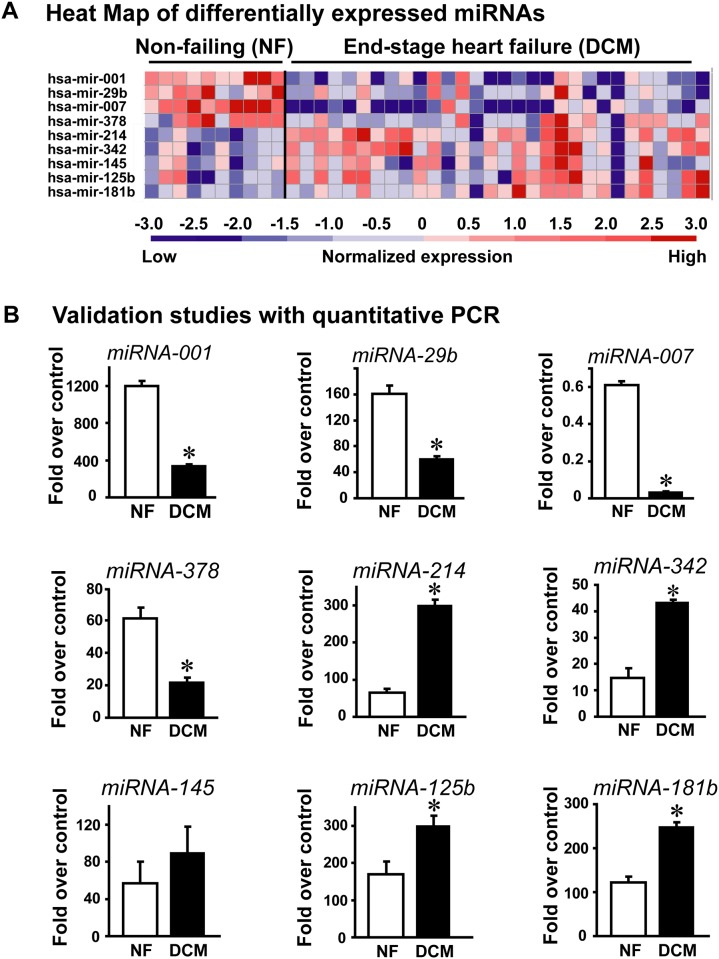
A, Heat map of significantly altered miRNAs from individual patient samples from non-failing human hearts and failing human hearts diagnosed with dilated cardiomyopathy. B, Semi-quantitative real time RT-PCR validation of the altered miRNAs identified by miRNA micro-array hybridization. cDNA synthesized from non-failing and DCM samples were subject to real time PCR using miRNA-001 specific primers along with U6 as internal control. Data are presented as fold over normalized internal RNU6B control. Similar method as described for miRNA-001 was used with specific primers for miRNA -29b, -007, -372, -214, -342, -145, -125 and -181b. *p< 0.00001 (miRNA-001, DCM vs. NF); *p< 0.0002 (miRNA-29b, DCM vs. NF); *p< 0.00007 (miRNA-007, DCM vs. NF); *p< 0.001 (miRNA-378, DCM vs. NF); *p< 0.0005 (miRNA-214, DCM vs. NF); *p< 0.003 (miRNA-342, DCM vs.NF); *p< 0.01 (miRNA-125b, DCM vs. NF); *p< 0.005 (miRNA-181b, DCM vs. NF).

The miRNA microarray hybridization was carried using 10 non-failing and 30 DCM human heart samples. The hybridization data from quantitative expression was normalized using global median and Lowess normalization (see Methods) wherein each patient sample was independently hybridized to generate expression profile. Following the iterative process of normalization, expression of nine miRNAs were found to be significantly different in DCM samples compared to non-failing human heart samples. The miRNAs *hsa*-mir-001 (p<0.00005), *hsa*-mir-29b (p<0.0087), *hsa*-mir-007 (p<0.00086) and *hsa*-mir-378 (p<0.0055) were significantly down-regulated in the DCM samples compared to non-failing controls. In contrast, miRNAs *hsa*-mir-214 (p<0.0001), *hsa*-mir-342 (p<0.0004), *hsa*-mir-145 (p<0.009), *hsa*-mir-125b (p<0.078) and *hsa*-mir-181b (p<0.0047) were significantly upregulated in DCM compared to non-failing controls. Despite the individual patient variability to each of the miRNA probes, the heat map shows distinctive pattern for specific miRNA expression associated with human DCM (Fig 1A).

To validate the expression pattern of these altered miRNAs, RT-PCR was performed on an independent set of patient samples containing 10 non-failing and 20 DCM samples using 18S and U6 as internal and the data has been normalized to U6 due to consistency in the U6 values across samples. RT-PCR on the new set of human samples showed that differential expression of all the identified miRNAs could be validated except *hsa*-mir-145. To further confirm these results, we also carried out RT-PCR on the set of samples used for the miRNA microarray study. Consistently, these studies also showed similar differential expression of miRNAs except for *hsa*-mir-145. The RT-PCR data from the two independent sets of human samples consistently showed the similar results and were pooled for analysis that includes 20 non-failing and 50 dilated cardiomyopathy human patient samples (Fig 1B). In addition to identifying miRNAs previously reported to be altered in human heart failure or mouse models of heart failure, our profiling with the custom miRNA microarray identified a novel miR, *hsa*-miRNA-7 to be significantly down regulated in DCM samples. Thus, using a large cohort of patient samples, our studies have identified the presence of a unique miRNA profile in end-stage DCM that now allows us to build signaling networks based on these altered miRNAs.

### Altered miRNAs are associated with specific canonical and functional pathways

Since the altered miRNAs modulate expression of respective targets simultaneously, the manifestation of the cardiac pathological phenotype/function is therefore a consequence of the sum total of effects coordinated by individual miRNAs. Insights into the understanding global mechanisms underlying heart failure pathology is critical given that multiple mRNA transcripts can be targeted by the same miRNA and alternatively a single mRNA transcript can be targeted by multiple miRNAs. To assess for the potential alterations in the global signaling networks that could occur in response to different molecules being simultaneous targets for the altered miRNAs, all the predicted targets for miRNA signature identified in our DCM patients were utilized for pathway analysis. To evaluate functional consequences based on combinatorial effect of the predicted targets of miRNAs an unbiased computational approach (see Methods) was taken using Ingenuity Pathway Knowledge base. The predicted target proteins based on their individual identified functional roles in signaling mechanisms and pathways are integrated into the Knowledge base algorithm that was used for determining the signaling/functional network. Analysis shows that a total of 1875 genes are predicted targets for the eight altered miRNAs identified in our study and were used for our computational approach. Use of these predicted targets with Ingenuity Pathway analysis identified that the most overrepresented canonical functional network with highest level of significance to be cardiovascular system development and function (Table 2). Identification of this network is entirely consistent with a role of the predicated targets of the altered miRNAs in cardiovascular disease. Further analysis of the molecules that are network eligible showed significant representation of the miRNA targets in cellular and molecular function including cell signaling, gene expression, cell death (Table 3). Identification of these functions indicates the rigor of the computational analysis as molecules in these processes are already well documented to be dysregulated in end-stage heart failure [25, 26]

**Table 2:** Physiological systems potentially affected by altered miRNAs in end stage heart failure (DCM) patient samples.

Molecular and Cellular Functions	p-value	# Molecules
**Cell Signaling**	**5.91E-146**	**368**
**Gene Expression**	**1.67E-126**	**254**
**Cell Death**	**4.55E-93**	**245**
**Cellular Growth and Development**	**8.94E-84**	**273**
**Cellular Development**	**6.71E-77**	**229**

**Table 3:** Molecular and cellular functions potentially targeted by altered miRNA in DCM samples.

Molecular and Cellular Functions	p-value	# Molecules
**Cell Signaling**	**5.91E-146**	**368**
**Gene Expression**	**1.67E-126**	**254**
**Cell Death**	**4.55E-93**	**245**
**Cellular Growth and Development**	**8.94E-84**	**273**
**Cellular Development**	**6.71E-77**	**229**

### Predicted targets of altered miRNAs associate with diverse signaling networks

Assessment of the 1785 predicated targets showed that 1716 molecules could be mapped to signaling networks (see Methods for definition of network) in the Ingenuity Pathways Knowledge Base network algorithm (IPA^™^). Off the 1716 molecules, 995 predicted targets were found to be network eligible (see Methods for eligibility). The 995 network eligible candidates mapped to 43 networks which are predicted to be involved in the cross-talk with the peripheral molecules bridging different networks (S2 Fig). The molecular cross-talk that occurs between all these networks ultimately results in the global effects that underlie/determine the DCM phenotype. A bio-informatic view of the global effects potentially mediated by the eight altered miRNAs on signaling in DCM is shown in (S2 Fig). This analysis indicates that global network analysis for a cardiac pathology can be built using the knowledge base algorithm that can either be scaled up or down based on the representative miRNA signature containing the most significantly altered miRNAs.

**Fig 2:**
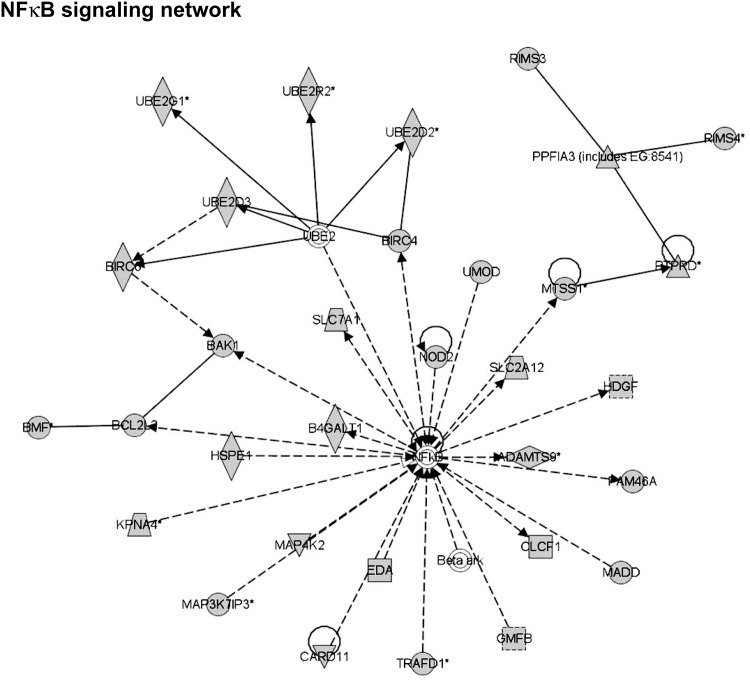
A representative network of molecules which are predicted targets of miRNAs 1, 214, 342, and 378. NF kappa B is the central nodal molecule of this network, but importantly, NF kappa B is not a predicted target to any of the altered miRNAs. The NF kappa B could be involved in pathogenesis as the molecules in the network are modulated by miRNAs, and the network function would change as a consequence. The solid arrow between molecules depicts experimentally proven relationship. A dashed line depicts inferred interaction based on experimental evidence. Solid feed-back circular line with an arrow (Auto-regulation). Representation of the shapes: Vertical Diamond–Enzyme; Horizontal Diamond–Peptidase; Equilateral Triangle–Phosphatase; Inverted Equilateral Triangle–Kinase; Solid line square–Cytokine; Dashed line square–Growth Factor; Circle–Other; Trapezoid–Transporter; Double line circle–Complex (like Beta ark).

A representative network with NF kappa B, a known mediator in cardiac dysfunction [27] as a central node, is shown in Fig 2 wherein many of the network members that feed into NF kappa B regulation are targets for the miRNAs 1, 29b, 125b, 181b 214, 342 and 378. As individual miRNA acts on each target, the net effect on the node would be the collective inputs of all the members that feed into the central node of NF kappa B (Fig 2). Based on our data that majority of these miRNAs (miRNAs 125b, 181b, 214 and 342) are upregulated in DCM indicating that they would reduce the expression of the targets, we predict that the NF kappa B regulatory signaling network (Fig 2) would be downregulated in DCM. Although Fig 2 predicts one specific network with NF kappa B as a central node for various miRNA, it is important to note that each individual network does not affect a physiological process in isolation as global regulation involves integrative cross-talk among the networks to mediate the disease phenotype. In order to test for such a cross-talk between networks, we have used Ingenuity Pathways Analysis algorithm to overlay and merge the connecting cross-talking networks to develop a global network signature that would predictably be altered in response to specific miRNA changes (S2 Fig). Further analysis of networks involved in top molecular and cellular functions associated with DCM (Table 3) showed that out of the 75 networks, only 42 are predicted to be involved in the cross-talk with the peripheral molecules of each individual network bridging/linking the neighbouring networks (S2 Fig). A dendrogram representation of a subset of merged networks (S3 Fig) predicts a set of represented pathways specific for DCM. Additionally, a part of the dendrogram in S3 Fig is presented in a nodal network format to provide an appreciation of the networking connections and potential regulatory mechanisms that could exist in a network cross-talk between various molecules (S4 Fig). The open ellipse in S3 and S4 Figs could be used as a sentinel for comparison between them. Taken together this iterative analysis indicates that a specific set of pathways are operational which are associated with integrative connected networks that results in the manifestation of DCM phenotype. Importantly, these networks are generated based on the altered pattern of miRNAs.

We next analysed whether nodes (which are key central molecules in networks to which all the peripheral molecules connect and regulate (see Methods) of the various networks which are interconnected and overrepresented in DCM are predicted targets for the differentially expressed miRNAs in end-stage heart failure. IPA predicted nodal molecules on the various merged networks were analysed for miRNA targets using the target prediction algorithms (see Methods). This analysis shows that a large number of nodal molecules are predicated targets of miRNAs identified in our DCM study (Table 4). Consistent with the understanding of the miRNA biology, some of the predicted molecules are potential targets for multiple miRNAs while some are targets for none. Since we have identified these nodal molecules in signaling networks that are constituted by over represented signaling pathways (Table 3), we assessed whether these nodal molecules are altered in end-stage human dilated cardiomyopathy using the publicly available cardio-genomics expression database (see Methods). Indepth analysis of the expression pattern of the nodal molecules from the cardio-genomics expression database showed that many of the nodal molecules are significantly altered in end-stage heart failure (Table 5, Green and Red represent down- and up- regulation respectively). Similarly, we have also mapped the changes in expression pattern of the miRNAs identified in our DCM study (Table 5, Green represents down-regulation and Red represents up-regulation) which now allows us to relate the expression pattern of the predicated target nodal molecule to the altered miRNA. Indeed, the data shows inverse co-relation in the expression level of the predicated target nodal molecules compared to the altered miRNAs identified in our DCM profiling like MLL, STAT3, ERBB2 (Table 5). Interestingly, we also see parallel alterations for some of nodal molecules and miRNAs e.g., MMPs, TIMP2 (Table 5) and in some cases no effect on the nodal molecule expression despite being predicted targets like RB1, EZH2 (Table 5). These observations suggest the miRNA mediated expression of molecules is one component of the complex pathophysiology of heart failure.

**Table 4:** Potential targets of validated miRNA occupying central nodes on networks.

Merged Networks							
1,2,31,32,89,9		11,15,2,21,32,36		17,20,22,32,7,8		27,28,31,33,39,42,44	
Nodal Molecule	Targeting hsa-miRNA	Nodal Molecule	Targeting hsa-miRNA	Nodal Molecule	Targeting hsa-miRNA	Nodal Molecule	Targeting hsa-miRNA
**MLL 342**	**342**	**PDGFRB**		**MBD2**	**7,125b**	**YWHAG**	
**RB1**	**7**	**SP1**	**3,378**	**RARB**	**1,29b**	**DUB**	
**CTBP1 &-2**		**PDGFRA**		**EZH2**	**378**	**SMARCA4**	
**STAT3**	**125b**	**STAT3**	**125b**	**E2F3**	**342,378**		
**E2F3 7**	**342,378**	**RB1**	**7**	**PER1**	**214**		
**Histone 3**	**1**	**BCL2**	**181b**	**CSF1**	**214**		
**MITF**	**378**	**ERBB2**	**7**	**CAMPK B,D**			
**NFkB**		**ACVR2B**	**214,18b**	**ZNF 217**			
**TERT**		**MMP2,11**	**125b**	**CCND1**			
**HDAC4 & 9**	**29b**	**TIMP2**	**214**	**CREB5**	**1,214,181b**		
**Col1A2**	**7,342**	**CDK6 PROTO-CADHER**	**214**				

**Table 5:** Expression of potential targets and validated miRNA in human cardiomyopathy.

Merged Networks											
1,2,31,32,89,9			11,15,2,21,32,36			17,20,22,32,7,8				27,28,31,33,39,42,44	
Nodal Molecules	Targeting hsa-miRNA		Nodal Molecules	Targeting hsa-miRNA		Nodal Molecules	Targeting hsa-miRNA			Nodal Molecules	Targeting hsa-miRNA
**MLL**	**342**		**PDGFRB**	**-**		**MBD2**	**7**	**125**		**YWHAG**	**-**
**RB1**	**7**		**SP1**	**7**	**378**	**RARB**	**1**	**29b**		**DUB**	**-**
**CTBP1 & 2**	**-**		**PDGFRA**	**-**		**EZH2**	**378**			**SMARCA4**	**1**
**STAT3**	**125b**		**STAT3**	**125b**		**E2F3**	**342**	**378**			
**E2F3**	**342**	**378**	**RB1**	**7**		**PER1**	**214**				
**HISTONE3**	**1**		**BCL2**	**181b**		**CSF1**	**214**				
**MITF**	**378**		**ERBB2**	**7**		**CAMK B**	**-**				
**NFkB**	**-**		**ACVR2B**	**214**	**181b**	**ZNF 217**	**-**				
**TERT**	**-**		**MMP2&11**	**125b**		**CCND1**	**1**				
**HDAC 4&9**	**29b**		**TIMP2**	**214**		**CREB5**	**1**	**214**	**181b**		
**COL1A2**	**7**	**342**	**CDK6**	**214**							
			**PROTO-CADHER**								

Green Highlighting = Down regulation; Red Highlighting = Up-regulation

### Analysis and validation of the miRNA targets and network

**Fig 3:**
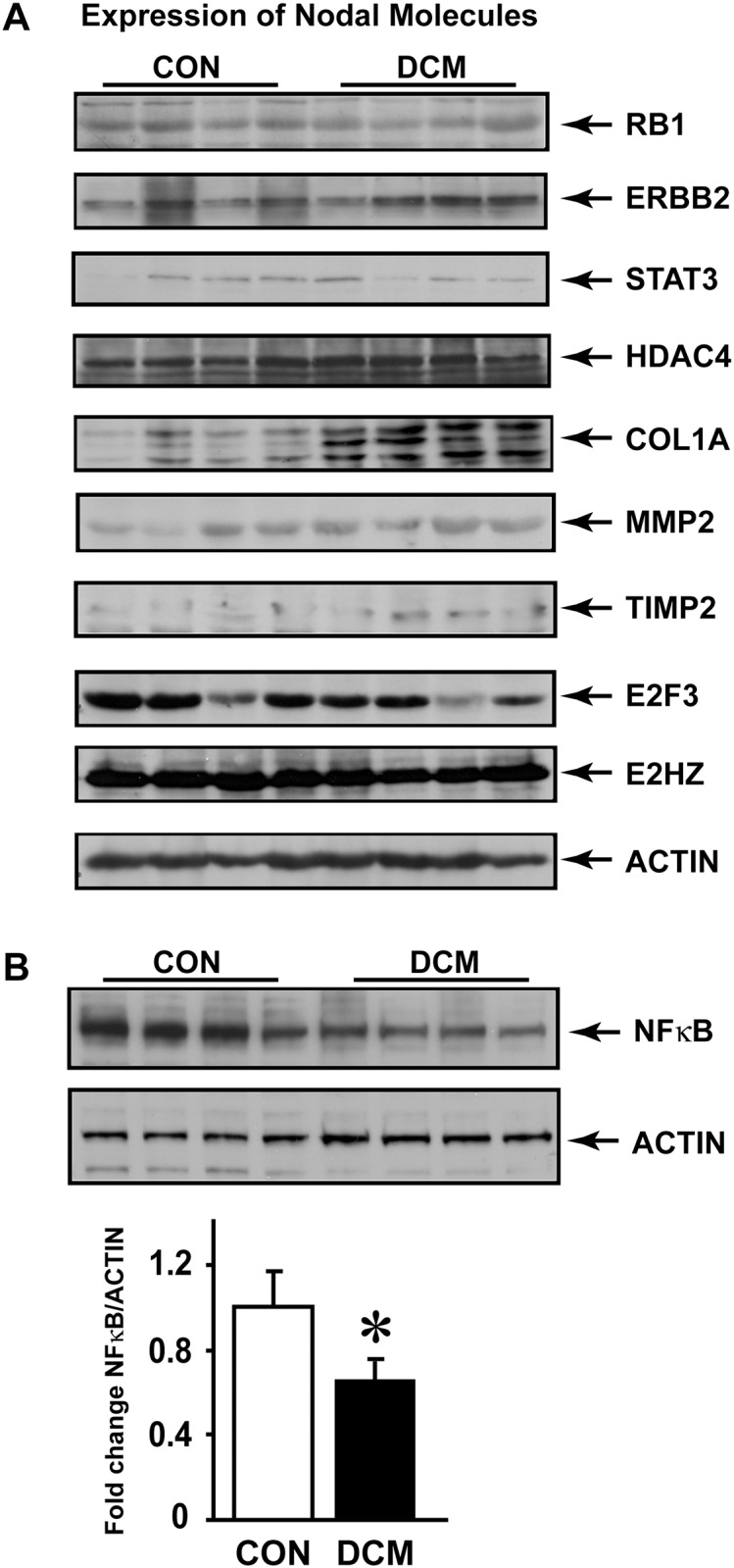
A, Western immunoblotting analysis was carried out non-failing human hearts (Controls, CON) and patients diagnosed with dilated cardiomyopathy (DCM). 170 μg of myocardial lysates were resolved with SDS-PAGE gel and immunoblotted with respective anti-bodies. beta-actin antibody was used for equal loading. B, Immunoblotting for nodal molecule NF kappa B was performed on non-failing human hearts (CON) and failing dilated cardiomyopathy (DCM) samples. Actin was used as loading control. Lower panel shows summary data, n = 8. *p< 0.05 DCM vs. CON.

To evaluate whether nodal molecules expression pattern inversely correlates with the altered miRNAs, western immunoblotting was carried out on end-stage DCM human heart failure samples. Based on the mechanisms of miRNA regulation of expression, ideally nodal molecules that are targets for miRNAs would have inverse co-relationship between miRNA levels and target nodal molecule expression. Indeed, western analysis of some of the nodal molecules well known to have a role in heart failure show inverse co-relationship with the level of respective miRNAs (Fig 3). Importantly, these observations are consistent with data in the publicly available cardiogenomics database (Table 4). Immunoblotting shows that ERBB2, COL1, MMP2 and TIMP2 have increased expression in human DCM (Fig 3) compared to non-failing and is inversely correlated to the downregulation of their respective miRNAs. In contrast, STAT3, and E2F3 seem to have decreased expression in the DCM samples consistent with the observation of upregulation of their respective miRNAs suggesting that expression of these molecules may be regulated by miRNAs. Since the network containing NF kappa B as a nodal molecule was used as an example, we assessed NF kappa B expression in non-failing and DCM end-stage human heart failure samples. Immunoblotting studies showed significant reduction in expression of NF kappa B in end-stage DCM samples compared to non-failing controls (Fig 3B, lower panel summary data, n = 8). These observations are consistent with the expression analysis observed in the Cardiogenomics data base (Table 4). Interestingly, we did not observe appreciable changes in expression levels of HDAC4, RB1 or EZH2 (Fig 3) despite being predicted targets for the altered miRNAs (Table 4).

**Fig 4:**
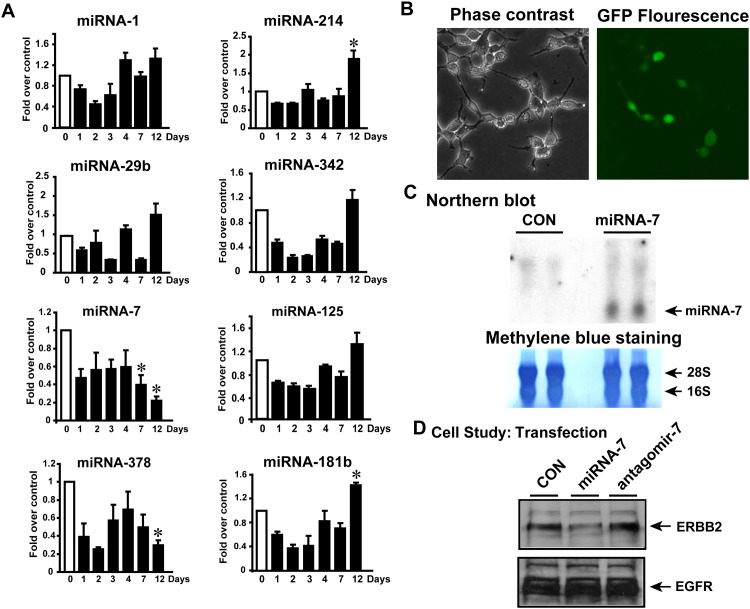
A, C57BL/6 mice underwent transverse aortic constriction (TAC) surgery and the experiment terminated on days 0 (sham), 1, 2, 3, 4, 7 and 12 post-TAC. This model of heart failure is known to initiate cardiac dysfunction by 12 days of TAC that is associated with cardiac hypertrophy. RNA was isolated from the excised hearts and subjected to qRT-PCR for the altered miRNAs identified in our study on end-stage heart failure. *p<0.05 vs. the control (sham) (n = 4). B, Phase contrast and confocal microscopy showing expression of miRNA7 transfected in HEK 293 cells. miRNA-7 encoding plasmid co-expresses green-fluorescence protein (GFP) to visualize transfection. C, To test for expression of miRNA7, northern blot analysis was carried. Con, represents vector transfected cells, miRNA7, represents cells expressing miRNA-7. Lower panel shows methylene blue staining of the nylon membrane depicting equal loading of RNA for each of the samples for northern analysis. D, miRNA-7 expressing cells were lysed and western immunoblotting carried out for nodal molecule target ERBB2. Interestingly, EGFR is not altered post-miRNA7 expression.

A key question from the above studies is whether these eight miRNAs that we have identified from the patient DCM tissues are the result of the cardiac pathology *ie* “effect” or whether these are integral contributors to the development of pathology *ie* “cause”. To determine their role in cardiac pathology, we used a mouse model of pressure-overload induced heart failure by performing transverse aortic constriction (TAC). TAC surgery was performed on C57Bl/6 mice and the experiment was terminated on 1, 2, 3, 4, 7 and 12 days post-TAC while mice with sham operation were considered as 0 day control. RNA isolated from the mouse hearts was subjected to real time PCR (RT-PCR) to evaluate the alteration in the 8 identified miRNAs. RT-PCR analysis of the 8 miRNAs showed that early in TAC *ie* for the first 3 days, all miRNAs are uniformly have decreased expression compared to the sham controls which may not be surprising given that the heart is recovering from surgery. Also, we did not observe any specific trends till day 7 wherein miRNA 7 showed consistent downregulation. Twelve days post-TAC (a time point known characterized by deleterious cardiac remodeling and hypertrophy [28] significant alteration in a subset of 4 miRNAs (miRNA 7, 378, 214 and 181b) were observed (Fig 4A), out of the 8 altered miRNAs identified by our DCM profiling studies (Fig 1). Importantly, miRNA 7 and 378 are significantly downregulated while miRNA 214 and 181b are significantly upregulated (Fig 4A) 12 days post-TAC. These studies show that with initiation of deleterious cardiac remodelling, a subset 4 miRNAs (out of a set of 8 miRNAs identified in our DCM studies) are altered and could play a role in regulating signaling networks contributing towards to global cardiac pathology.

Although TAC alters the expression of these miRNAs, to validate expression of targets observed in DCM samples, we used miRNA 7 in our experimental studies as it is 1) a novel miRNA identified in our profiling, 2) it targets EGFR growth family receptor ERBB2, a critical player cardiac hypertrophic response [29, 30] and is one of the miRNAs down regulated early post-TAC. Expression of miRNA 7 constructs were assessed by confocal microscopy (Fig 4B) and northern blot analysis (Fig 4C) following transfection of HEK 293 cells. To directly test for dynamic regulation of ERBB2 expression by miRNA 7, HEK 293 cells were transfected with miRNA 7 or its antagomir to assess ERBB2 expression. Consistently, expression of miRNA 7 results in decrease in ERBB2 expression which is markedly reversed following antagomir 7 (Fig 4D, upper panel). Importantly, we observed no changes in the EGFR expression suggesting specific targeting of ERBB2 by miRNA 7 (Fig 4D, lower panel).

**Fig 5:**
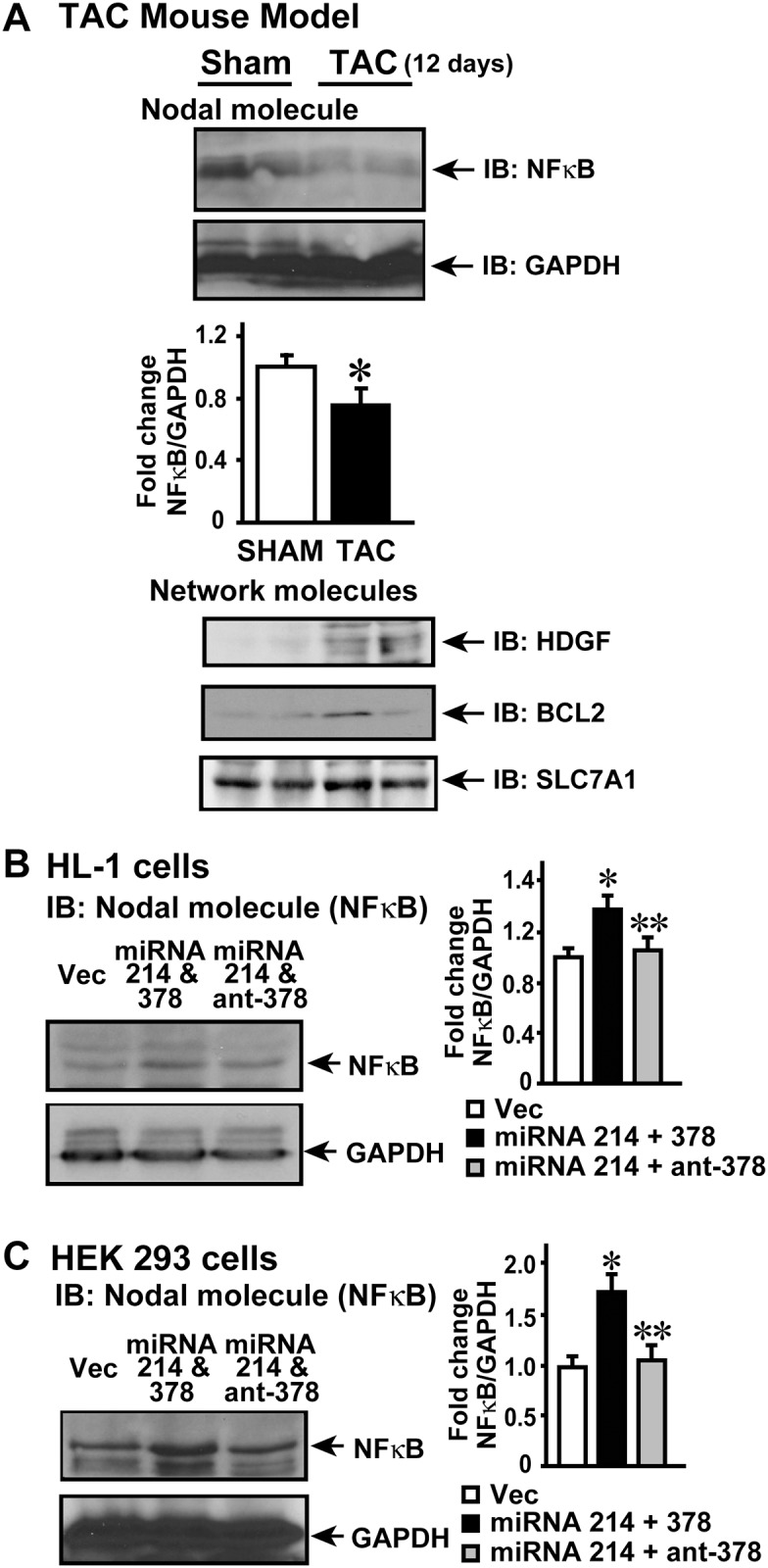
A, Since miRNA214 is upregulated and miRNA 378 is downregulated following 12 days of TAC, western immunoblotting was carried out for nodal molecule (NF kappa B) and other network molecules (HDGF, BCL2 and SLC7A1). GAPDH was used a loading control, *p<0.05 vs. Sham, (n = 8). B, HL-1 cardiomyocyte cells were transfected with allstar scrambled control (Vec), or miRNA 214 along with miRNA 378 or miRNA 214 along with antagomir for miRNA 378. The cells were lysed and subjected to immunoblotting for NF kappa B. GAPDH was used as loading control. *p<0.05 vs. Vec (scrambled vector control transfected cells); **p<0.05 vs. miRNA 214 & 378 transfected cells (n = 3). C, HEK 293 cells were transfected with Vec or miRNA 214 along with miRNA 378 or miRNA 214 along with antagomir for miRNA 378. The cell lysates were subjected to immunoblotting for NF kappa B. GAPDH was used as loading control. *p<0.05 vs. Vec; **p<0.05 vs. miRNA 214 & 378 transfected cells (n = 3).

These above studies provide a glimpse of a single miRNA regulation of a specific target, but however as we are assessing the effect of miRNAs on a network, we tested whether using a combination of miRNAs/antagomirs would alter NF kappa B signaling network (Fig 2). NF kappa B signaling network has 35 molecules including NF kappa B. Analysis of the predicted targets indicated that 22 molecules of the NF kappa B signaling network are targets for the miRNAs altered in our DCM studies. Since 4 of the miRNAs from our DCM studies are altered early in TAC, we assessed the predicted targets of these 4 miRNAs in the NF kappa B signaling network. Analysis of the predicted targets for the 4 miRNAs indicated that NF kappa B signaling network has 12 molecules that are targets for miRNA 378, 214 and 181b. Predicted targets for altered miRNAs are shown in S1 Table. HDGF and BCL2 are predicted targets for miRNA 214, while CLCF1 and SLC7A1 are predicted targets for miRNA 378 indicating that these molecules may feed into the network with initiation of cardiac stress resulting in modulation of NF kappa B which is not a target of either miRNA 214 or 378. Although CLCF1 is a predicted target of miRNA 378, its expression pattern did not inversely correlate with miRNA expression profile indicating that it may be regulated by mechanism(s) independent of miRNA 378. Furthermore, none of the predicted targets for miRNA 181b were altered in NF kappa B network. Since our 12 day TAC hearts showed that miRNA 214 and 378 are altered early, we assessed whether it could alter nodal NF kappa B as a part of change in network system. Immunoblotting showed significant reduction in NF kappa B expression following 12 days of TAC compared to sham (Fig 5A upper panel, middle panel summary data (n = 8). GAPDH was used as loading control. There was significant upregulation of HDGF following TAC (Fig 5A, lower panel) while we also observed concomitant increase in BCL2 though not as robust at HDGF (Fig 5A, lower panel). Upregulation of HDGF and BCL2 show that their regulation is independent of miRNA 214 because as predicted targets they should be down regulated since miRNA 214 is elevated post-TAC (Fig 4A). However, SLC7A1 is moderately upregulated following 12 days of TAC compared to sham consistent with it being a target miRNA 378 that is down regulated following TAC (Fig 4A). Given the caveat that following TAC many pathways may feed into NF kappa B regulating its expression, we used cardiomyocyte cell line HL-1 and HEK 293 cells to monitor acute effects on NF kappa B expression following miRNA and antagomir transfection. HL-1 cells were transfected with scrambled allstar control (Vector–Vec), miRNA 214 and 378 together (miRNA 214 & 378) or miRNA 214 and antagomir 378 (ant-378) together (to mimic the TAC scenario of elevated miRNA 214 along with concomitant reduction in miRNA 378). Following forty eight hours of transfection, cells were lysed and immunoblotted for NF kappa B. Consistent with our TAC studies, we observed significant reduction in NFκB expression following miRNA 214 and antagomir 378 (Fig 5B, left panel, right panel- summary data (n = 3) while GAPDH was used a control. This observation supports the idea that miRNA targeting of the network molecules could have a cumulative effect on the nodal molecule like NF kappa B which is not a direct target for these miRNAs. To further test whether such network regulation of nodal molecule could be conserved, HEK 293 cells were transfected with scrambled allstar control (Vector–Vec), miRNA 214 and 378 together (miRNA 214 & 378) or miRNA 214 and antagomir 378 (ant-378). Correspondingly, significant increase in NF kappa B was observed with expression of miRNAs 214 and 378 which is completely reversed by the expression of miRNA 214 and antoagomir-378 (Fig 5C, left panel, right panel- summary data (n = 3), and GAPDH was used a loading control. Taken together our studies using mouse model and cell system show that miRNAs mediate regulation of signaling network like the example of NF kappa B wherein, perturbation of peripheral molecules in the network by miRNA 378 seems to modulate expression of nodal molecule NF kappa B as predicted by IPA network analysis. These observations bring-to-fore a new layer of complexity involving cross-talk between various molecules based on their location in the network and how they are in turn regulated by changes in the miRNA expression pattern.

## Discussion

Mechanisms that alter global signaling networks underlying endstage DCM in humans are largely unknown. In this study, we have utilized the unique miRNA signature observed in the end-stage human heart failure DCM samples to define global molecular signaling networks regulated by miRNAs that identifies the cross-talk between molecules fundamental to the overall DCM phenotype. Using a relatively large set of human samples and custom miRNA microarray platform, based on our cut-off we have identified 8 differentially expressed miRNAs in DCM compared to non-failing human hearts. In addition to identifying known miRNAs that are dysregulated in heart failure [31], we also identified a novel miRNA, miRNA 7which has not yet been implicated in cardiac pathophysiology. The signaling networks and functional pathways formed by the predicted targets of these altered miRNAs enabled an unbiased prediction of molecular cardiovascular disease bio-function in understanding heart failure biology within the Ingenuity^™^ knowledge compendium. Critically the use of the miRNA profile allowed us to identify nodal molecules on the global signaling networks that could be altered in response to miRNA targeting of the peripheral molecules laying the foundation for us to propose a global regulatory role of miRNAs in modulating molecular networks. A key idea that our study shows is that the nodal molecules in the signaling network need not be direct targets for the miRNAs but could still be regulated due alterations in the neighbouring molecules due to miRNA targeting within the network. To further support this idea, we have validated the expression pattern of nodal molecules following meta-analysis of heart failure gene expression fingerprint from the publicly available cardio-genomics database. Validation performed by immunoblotting on some of the nodal molecules further confirms the data from the cardio-genomic database. Finally, we have used TAC mouse model and HL-1 cells to show the regulation of the NF kappa B nodal molecule and the inputs into its regulation by neighbouring miRNA targets within the NF kappa B signaling network.

Our custom made miRNA microarray and RT-PCR validation from an independent set human heart failure samples identified eight miRNAs to be differentially expressed in human DCM. Importantly, along with identification with miRNAs that have been previously reported in mice and human studies [5, 32, 33]. Our current study has identified a novel miRNA (miRNA -7) which is differentially expressed in DCM due to the miRNA micro array being custom made as supposed to those available commercially. Another key strength of our study is the use of large numbers of heart failure samples that adds to the rigor of our miRNA microarray. Since we have used end-stage heart failure samples, a key question that arises is whether these top altered miRNAs are the “cause” or an ‘effect” off heart failure that occurs due to cumulative deleterious cardiac remodeling. To address this question we used the TAC mouse model to test for alteration in miRNAs with initiation of cardiac dysfunction. Off the eight miRNAs altered in human DCM patients, we observed only a subset of 4 miRNAs (miRNAs 7, 378, 214 and 181b) to be altered with initiation of cardiac dysfunction suggesting that other miRNAs identified in the study may be an effect of heart failure. Further indepth studies are required to determine the role of these miRNAs in cardiac remodeling with initiation of stress.

Our study identified miRNA 7 which until now has not yet been implicated in cardiac pathophysiology. miRNA 7 is downregulated in end-stage DCM as well as early in TAC that could result in upregulation of its targets in cardiac pathology. Predicted targets of miRNA-7 encompass many of the key nodal molecules in merged networks including ERBB2 (epidermal growth factor receptor 2) and collagen 1 (COL1A) that are known to play key roles in cardiac remodeling. Consistently, western blot analysis from the DCM samples shows significant upregulation of miRNA -7 specific nodal molecules like ERBB2 and COL1A without changes in RB1 suggesting the ERBB2 and COL1A are targets for miRNA-7. Indeed, overexpression of miRNA-7 in cells shows significant downregulation of ERBB2 but not EGFR1 (Fig 4D) in contrast to EGFR being targeted by miRNA 7 in cancer cells [34, 35] suggesting cell specific regulation of miRNA targeting the EGFR receptors. Importantly, analysis of some of the predicated targets of miRNA-7 like Phospholipase Cβ (PLCβ), regulator of G-protein signaling (RGS), RAF1, phosphodiesterase 4 (PDE4) which are well studied in heart failure [24–26] are all upregulated in cardiac hypertrophy and heart failure [36–38] consistent with downregulation of miRNA 7. Studies on neuronal development have shown that miRNA 7 targets basic helix-loop-helix (HLH) and Brd box [39, 40] containing transcriptional repressor proteins that interpret notch signaling. Whether such regulation exists in cardiac system needs further elucidation. These studies suggest an important role for miRNA 7 and the role it plays in heart failure pathology is currently an active area of our study.

The miRNA-mediated regulation of gene expression within myocardium is perhaps one of the most intriguing mechanisms in heart failure biology. Although there are several studies that have defined the role of various miRNAs in heart failure [31], a key component that has not been addressed in these studies is the fact that these miRNAs are all altered simultaneously. Therefore, their effects on gene expression would be parallel and concomitant mediating the overall cardiac phenotype. Since miRNAs regulate expression mostly through repression, a set of expressed miRNAs would selectively target specific molecules in a combinatorial manner bringing about profound system wide influence on global gene expression programs. Thus, combinatorial expression patterns of miRNAs associated with DCM provides the mechanistic basis for reading out cooperative action of miRNAs on gene expression in human heart failure. In this context, traditional molecular profiling analyses have focussed on identifying individual genes deregulated during the disease process, but fail to show overlap between individual genes and processes the underlie the cross talk driving heart failure. Given this complexity, we moved beyond the single-miRNA or single gene target approach and analysed our target-scan signatures using IPA which allows for identification of enriched networks. The “targets of altered miRNA in heart failure” uncovered the GO process containing cardiovascular development and reprogramming. Further analysis identified networks of 1, 2, 31 and 32 to name a few as potential targets of miRNA regulation. One of these networks, network # 1 was the most enriched for genes with defined NF kappa B binding sites in their proximal promoters. These results suggest that a major process affected by miRNA regulation in network # 1 associated with heart failure progression is probably through NF kappa B target genes. This is consistent with growing body of evidence showing that inhibition of NF kappa B significantly ameliorates cardiac dysfunction by reducing pro-inflammatory responses [41–43]. However, it needs to be appreciated that NF kappa B is not regulated by a single mechanism either for its activation or expression. This may be key underlying reason for variability in expression of NF kappa B given its key role and redundant mechanisms of regulation. In addition, it is important to note that the variability in NF kappa B expression could be due to differential underpinning etiology prior to the clinical identification as DCM in human heart failure. Similarly, even in mice we observed variability in expression of NF kappa B in the heart post-TAC due to variation in generation of trans-stenotic pressure gradient with TAC that underlies the cardiac remodeling. Perhaps such variability indicates a key role for NF kappa B in cardiac remodeling given that its expression patterns can be potentially sense the level of cardiac stress. Our studies have been focussed on expression and not activation as miRNA driven mechanisms classically alter expression patterns. Although expression NF kappa B may be ~ 15–20% lower in conditions of cardiac stress but this does not reflects its activation as it is well known that NF kappa B is significantly activated in mediating responses of cardiac remodeling to mechanical overload/cardiac stress.

Evidence from several studies have showed the involvement of multiple miRNAs in regulation of hypertrophy and heart failure [5, 32, 33] suggesting global regulation of signaling networks leading to the ultimate physiological responses. Indeed studies on cardiac specific deletion of dicer support the idea that miRNAs globally regulate cardiac signaling and function as loss of dicer leads to DCM and heart failure [35]. Since the knowledge of underlying networks and canonical function pathways regulated by the miRNAs in conditions of heart failure is not known, we have examined potential regulation of specific signaling networks by differentially expressed miRNA in heart failure. Analysis of the predicated targets of differentially expressed eight miRNAs using the Ingenuity Pathways Network algorithm showed that these molecules are associated with network that defines cardiovascular development and function. Critically, this association demonstrates that by using an independent “in silico” methodology that these unrelated predicated targets for differentially expressed miRNAs co-relate to signaling components in cardiovascular system providing an algorithmic corroboration of our findings. This corroboration is further supported by immunoblotting of DCM samples showing inverse co-relationship between expression patterns of the predicted target nodal molecules to the alterations of their respective miRNAs. These observations strengthen the idea that miRNAs could regulate unrelated molecules in a signaling network that goes beyond the traditional feed-back or feed-forward loops. However, it is important to note that expression of not all the nodal molecules follow the inverse co-relationship with the altered miRNAs like RB1, HDAC4 or EZH2. There expression did not change despite significant changes in the miRNAs that target them suggesting mechanisms beyond the miRNA regulation. In this context, there is increasing appreciation for idea that there could be competing factors that regulate the miRNA binding to the RNA including RNA-binding proteins (RBPs) [44, 45] which in turn would negate the miRNA efficacy in binding and regulating RNA. Such an idea is supported by the unique RBPs identified in the cardiomyocytes and their role in regulating multiple cellular functions [46] and importantly would compete with the miRNA regulation of RNA globally as counter-balance.

Given this concept of global regulation, it is critical to determine regulation of signaling networks by miRNAs to provide an understanding of the global scale of regulation by miRNAs instead of assessing individual targets. As a proof-of-concept that miRNAs do regulate the signaling networks, we have analysed the predicted targets of miRNAs altered with initiation of cardiac dysfunction within the NF kappa B signaling network. Our data shows that perturbation of targets with miRNAs leads to modulation of network proteins that in turn alters the nodal molecule NF kappa B. Using HL-1 cells and TAC model, we show that nodal molecule NF kappa B which is not a predicted target for miRNA 378 is altered following acute as well as short terms changes in miRNA 378. These findings suggest that miRNAs will have a prominent role to play in altering global signaling networks and pathways in progression towards cardiac hypertrophy and failure. Also these observations supports the concept that miRNA targeting of specific molecules may have additional effects on the global phenotype due to the cross-talk inputs mediated by the role of the predicted targets in an active signaling network. Although our studies have focussed on miRNA mediated regulation of signaling networks it is possible that NF kappa B could still be regulated by other mechanisms in the TAC studies which is out of the scope of our current studies.

Despite the elucidation of the several clinically relevant signal transduction pathways that can lead to heart disease progression, the means by which these pathways are coordinated with respect to the development of cardiac dysfunction remain obscure. Manifestation of the phenotype of dilated cardiomyopathy is the net result of traditional cross-talk between the molecules and the non-traditional regulation by miRNAs. Inclusion of all the predicted targets for these altered miRNAs showed that they represent 75 annotated networks out of which 44 networks could be merged to cross-talk leading to global regulation of signaling. The cross-talk between the networks happen via the peripheral interconnecting molecules that are represented in between the networks (S2 Fig). Analysis of this kind provides for a global understanding of signaling networks and importantly sheds light on the atypical regulation of molecules by miRNAs resulting in a specific phenotype that cannot be explained by currently signaling paradigms. One of the major difficulties for functional studies of miRNAs is in determining their specific target genes as available algorithms predict hundreds of target genes for any single miRNA with a likely high fraction of false positives. Despite these deficiencies, use of all the predicted targets for the differentially expressed miRNAs with the network analysis algorithm shows that these molecules regulate cardiovascular system and cell signaling by an unbiased approach. Thus, our studies on global analysis suggest that networking biology approach may be a valuable tool that can be employed effectively to empower us in interpreting observation that do not fit into the classical paradigm of understanding. Our study therefore lays the foundation to the concept that targeting miRNAs would have profound global effects on signaling networks in a non-conventional manner compared to the conventional feed-back mechanism of regulation which needs to be appreciated as we understand and design better therapeutic approaches.

## Supporting information

S1 Table Molecules of NF kappa B signaling network that are predicted targets of altered miRNAs following 12 days of TAC. *hsa*-miR -125b, -214, -342, -181b (highlighted in red) are upregulated and *hsa*-miR -29b, -378 (highlighted in green) are downregulated. miRNA targeting of these network proteins could have a collaborative effect on nodal molecule NF kappa B despite NF kappa B not being a predicted target for altered miRNAs post-TAC.
(DOCX)

S1 Fig Schematic depiction of the experimental design used in the current study. See Methods for details of sample description.
(TIF)

S2 Fig Each square depicts a network. Off the potential 75 networks that are generated by the predicted targets, only 41 networks interact with each other in potentially modulating cardiac signaling. 27 networks do not interact with each other and therefore may not be integral to the global signaling networks that are potentially regulated by miRNAs. The number represented between the networks indicates number of molecules that could be involved in cross-talk between two networks.
(TIF)

S3 Fig A dendrogram illustration of merging of networks. Each network is made up of a group of molecules that are known to regulate and cross-talk with each other via a central nodal molecule. When networks are merged a large spider web is generated as shown here which represents networks 11, 15, 2, 22, 32 and 36. Similar spider web can be illustrated for all the 41 interacting networks in which there would be molecule(s) in one network that would cross-talk and regulate molecule(s) in another network.
(TIF)

S4 Fig Illustration representing merge of networks 11, 15, 2, 22, 32 and 36 showing specific gene positions for merged networks. The open ellipse represents a marker for comparing the dendrogram illustration to the functional gene-specific representation. The open ellipse represents EGFR as a nodal molecule in the network merge that could potentially have effects in cardiac remodeling which could be regulated by the neighboring molecules like integrin, PLC gamma, SOS, PDGFRB etc.
(TIF)
