# Supplementary material for: A unique microRNA profile in end-stage heart failure indicates alterations in specific cardiovascular signaling networks
Source: PLoS One. 2017 Mar 22;12(3):e0170456. doi: 10.1371/journal.pone.0170456 (PMC5362047; doi:10.1371/journal.pone.0170456)
Supplement: S1 Table — hsa-miR -125b, -214, -342, -181b (highlighted in red) are upregulated and hsa-miR -29b, -378 (highlighted in green) are downregulated. miRNA targeting of these network proteins could have a collaborative effect on nodal molecule NF kappa B despite NF kappa B not being a predicted target for altered miRNAs post-TAC. (DOCX) [file pone.0170456.s001.docx]

**Supplementary Table 1: Potential targets of altered miRNAs in NF-κB signaling network**

_________________________________________________

**Network Molecules Targeting miRNA(s)**

**_________________________________________________**

| MAP4K2 *hsa-mir 1* | |
| --- | --- |
| HSPE1 *hsa-mir 1* |  |
| FAM46A *hsa-mir 125b* |  |
| MADD *hsa-mir 125b* |  |
| BCL2L2 *hsa-mir 214* |  |
| UMOD *hsa-mir 214* |  |
| HDGF *hsa-mir 214* |  |
| MAP3K7IP3 *hsa-mir 214 & 378* |  |
| KPNA4 *hsa-mir 214 & 378* |  |
| BIRC4 *hsa-mir 218* |  |
| TRAFD1 *hsa-mir 29b* |  |
| ADAMTS9 *hsa-mir 29b* |  |
| EDA *hsa-mir 342* |  |
| MTSS1 *hsa-mir 342 & 1* |  |
| CLCF1 *hsa-mir 378* |  |
| SLC7A1 *hsa-mir 378* |  |
| NOD2 *hsa-mir 378* |  |
| SLC2A12 *hsa-mir 378* |  |
| BAK1  *hsa-mir 125b* |  |
| GMFB *hsa-mir 181b* |  |
| CARD11 *hsa-mir 181b* |  |
| B4GALT1 *hsa-mir 181b* |  |

**_____________________________________________________**

**Red – upregulation**

**Green - downregulation**
