# Supplementary figures and images for: A unique microRNA profile in end-stage heart failure indicates alterations in specific cardiovascular signaling networks
Source: PLoS One. 2017 Mar 22;12(3):e0170456. doi: 10.1371/journal.pone.0170456 (PMC5362047; doi:10.1371/journal.pone.0170456)

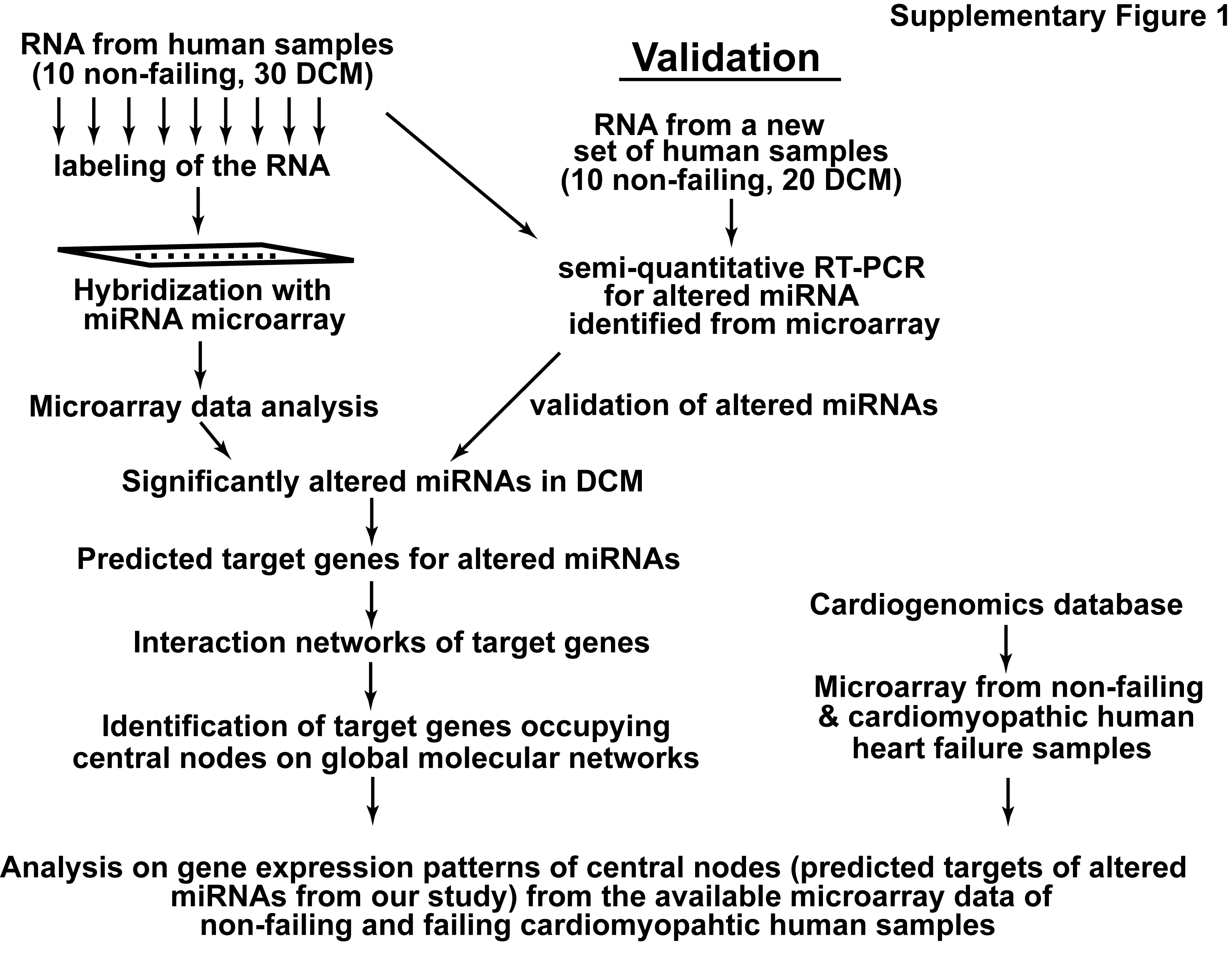

Supplement: S1 Fig — See Methods for details of sample description. (TIF) [file pone.0170456.s002.tif]

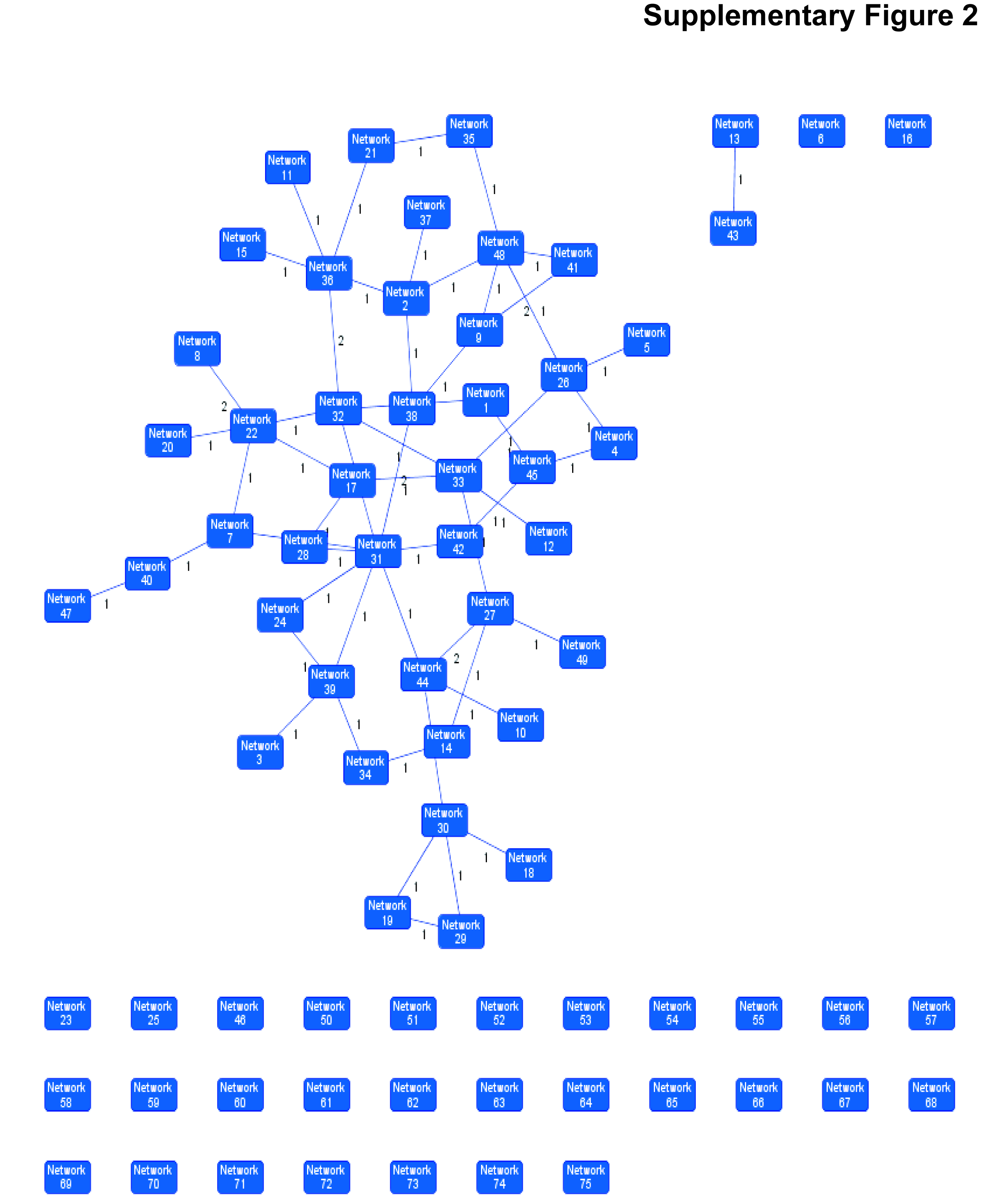

Supplement: S2 Fig — Off the potential 75 networks that are generated by the predicted targets, only 41 networks interact with each other in potentially modulating cardiac signaling. 27 networks do not interact with each other and therefore may not be integral to the global signaling networks that are potentially regulated by miRNAs. The number represented between the networks indicates number of molecules that could be involved in cross-talk between two networks. (TIF) [file pone.0170456.s003.tif]

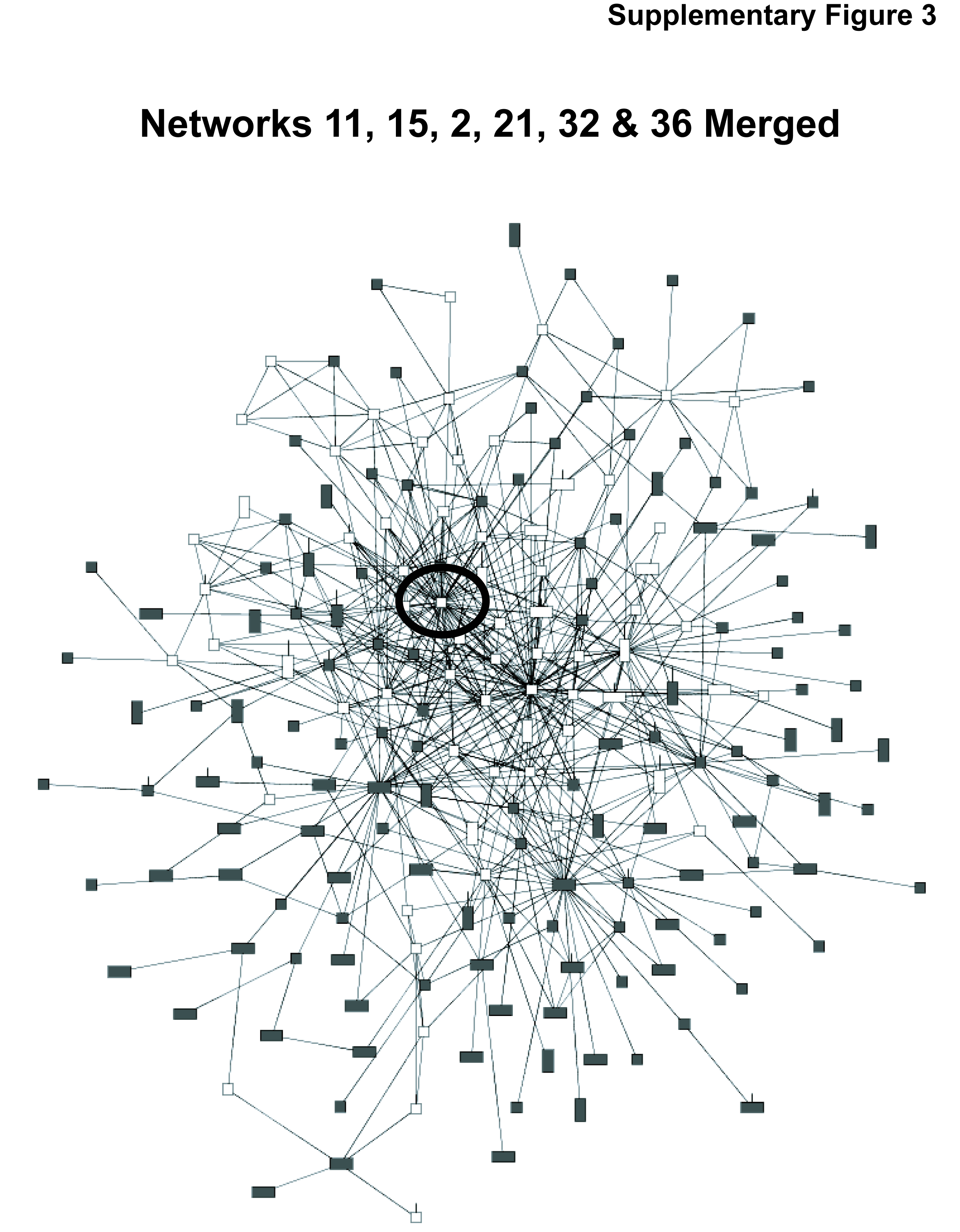

Supplement: S3 Fig — Each network is made up of a group of molecules that are known to regulate and cross-talk with each other via a central nodal molecule. When networks are merged a large spider web is generated as shown here which represents networks 11, 15, 2, 22, 32 and 36. Similar spider web can be illustrated for all the 41 interacting networks in which there would be molecule(s) in one network that would cross-talk and regulate molecule(s) in another network. (TIF) [file pone.0170456.s004.tif]

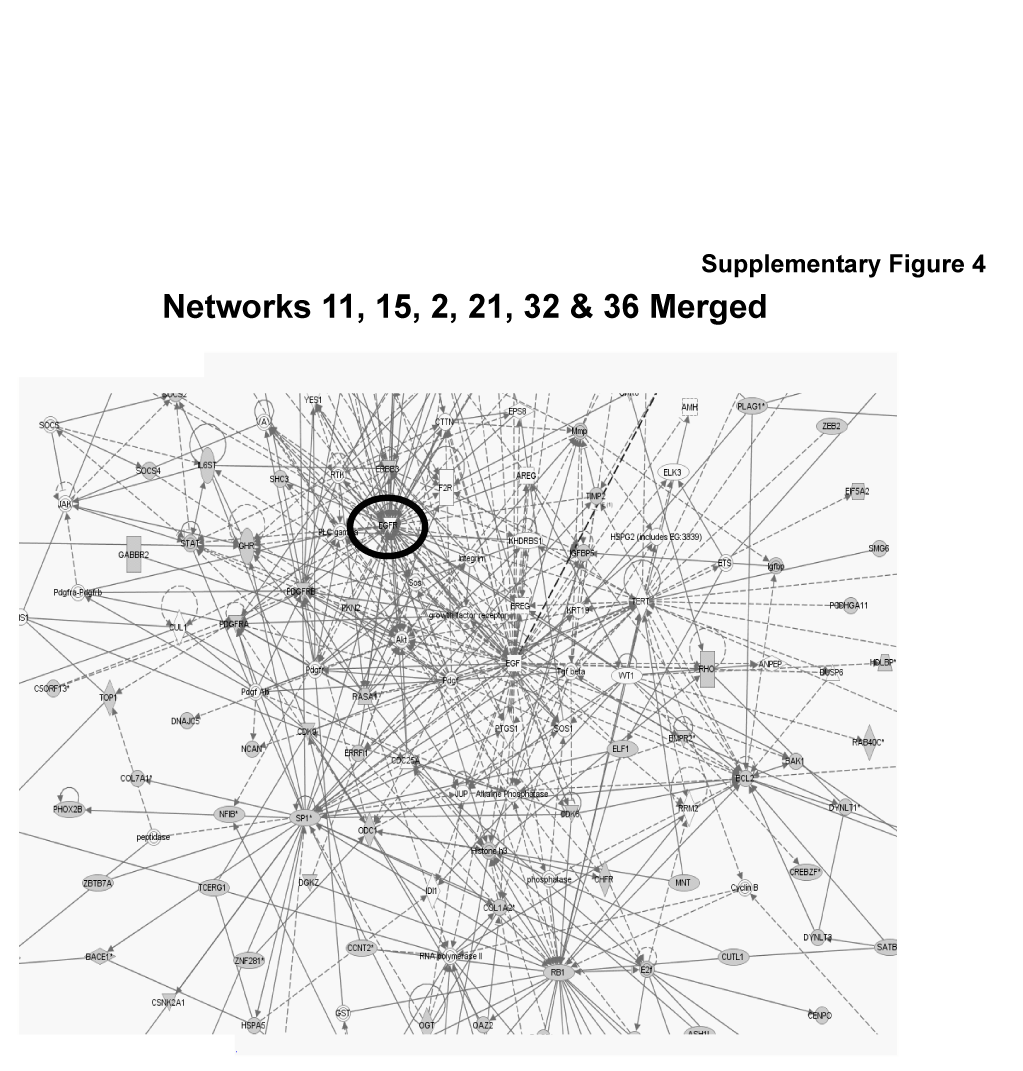

Supplement: S4 Fig — The open ellipse represents a marker for comparing the dendrogram illustration to the functional gene-specific representation. The open ellipse represents EGFR as a nodal molecule in the network merge that could potentially have effects in cardiac remodeling which could be regulated by the neighboring molecules like integrin, PLC gamma, SOS, PDGFRB etc. (TIF) [file pone.0170456.s005.tif]
